# Dispersive Liquid-Liquid Microextraction Followed by HS-SPME for the Determination of Flavor Enhancers in Seafood Using GC-MS

**DOI:** 10.3390/foods11101507

**Published:** 2022-05-22

**Authors:** Xiaolin Luo, Xiaoyuan Wang, Ming Du, Xianbing Xu

**Affiliations:** National Engineering Research Center of Seafood, School of Food Science and Technology, Dalian Polytechnic University, Dalian 116034, China; lxl1617010328@163.com (X.L.); xiaoyuanw122@163.com (X.W.); duming121@163.com (M.D.)

**Keywords:** dispersive liquid-liquid microextraction, HS-SPME, flavor enhancer, seafood, GC-MS

## Abstract

The determination of flavor compounds using headspace solid-phase microextraction (HS-SPME) combined with gas chromatography–mass spectrometry (GC-MS) can be severely interfered with by complex food matrices in food systems, especially solid samples. In this study, dispersive liquid-liquid microextraction (DLLME) was applied prior to HS-SPME to efficiently reduce the matrix effect in solid seafood samples. The method had high sensitivity (the quantification limits of maltol and ethyl maltol were 15 and 5 μg/kg, respectively), an excellent linear relationship (R2 ≥ 0.996), and the sample recovery rate was 89.0–118.6%. The relative standard deviation (RSD %) values for maltol and ethyl maltol were lower than 10%. Maltol (from 0.7 to 2.2 μg/g) and ethyl maltol (from 0.9 to 34.7 μg/g) in seafood were detected in the selected samples by the developed method. Finally, DLLME coupled with HS-SPME effectively removed the influence of sample matrix and improved the sensitivity of the method. The developed method was applicable in the analysis of flavor enhancers in complex matrix foods.

## 1. Introduction

Maltol and ethyl maltol, which are derived from sucrose pyrolysis and food baking, have a caramelized flavor, can enhance the flavor and sweetness of food [1,2], and are often used as food flavor enhancers in seafood processing to cover up a fishy smell and improve the flavor of products [3,4]. However, excessive consumption of flavor enhancers can have adverse effects on health, such as dizziness and nausea [5,6]. Meanwhile, maltol can chelate metal ions to form derivative complexes, which have certain toxicity to cells and affect liver and kidney function [7]. Studies had shown that when maltol was fed to rats at a daily dose of 1000 mg/kg, it caused kidney damage and even death [8]. Therefore, the amount of maltol and ethyl maltol added into the diet should be strictly monitored and an analytical method needs to be established that can improve detection sensitivity, and therefore, accurately detect and control the content of flavor enhancers in food samples.

As compared with the HPLC-MS method [9,10,11], the GC-MS method, with its specific advantages such as high separation resolution and reliable spectrum library search, has been mainly applied to determinate volatile compounds [12,13,14]. Headspace solid-phase microextraction (HS-SPME) has been a popular pretreat method applied to enrich volatile compounds for further quantitation and quantification using the GC-MS method [15,16,17]. Although the headspace method can effectively remove the nonvolatile compounds injected into a GC sample port [18], the enrichment of volatile compounds is severely interfered with by sample matrix, which consequently induces low sensitivity and less robustness for several volatile compounds [19,20]. Therefore, there is an urgent need to reduce the matrix effect of headspace methods for determining volatile compounds using the GC-MS method.

Traditionally, solid phase extraction (SPE) or liquid-liquid extraction (LLE) have been applied to remove most sample matrix [21,22], however, these traditional methods are time-consuming and consume large volumes of solvent [23,24]. As compared with traditional methods, the dispersive liquid-liquid microextraction (DLLME) technique can reduce solvent consumption and can concentrate analytes rapidly, which significantly improves the extraction efficiency [25,26,27]. In addition, DLLME had been reported to significantly reduce matrix interference in analyses of contaminants in wine [27,28], polycyclic aromatic hydrocarbons (PAHs) in roasted cocoa beans [29], and acrylamide in coffee samples [30]. To the best of our knowledge, there are no reports on HS-SPME combined with DLLME applied to detect flavor enhancers in seafood.

In this study, solid seafood was pretreated using DLLME/HS-SPME, and the flavor substances (maltol and ethyl maltol) in seafood were detected and analyzed by gas chromatography–mass spectrometry. The experimental parameters of DLLME and HS-SPME were optimized, and the accuracy and performance of DLLME/HS-SPME/GC-MS were further evaluated.

## 2. Materials and Methods

### 2.1. Chemicals

Maltol (99%) and ethyl maltol (99%) were purchased from Macklin (Shanghai, China). The analytical grade organic reagents dichloromethane (CH_2_Cl_2_), chloroform (CHCl_3_), carbon disulfide (CS_2_), methanol, acetone, and acetonitrile were from Sigma-Aldrich (St. Louis, MO, USA).

### 2.2. Preprocessing Method of DLLME/HS-SPME

Four types of seafood such as dried squid, instant squid larvae, instant kelp, and instant small yellow croaker were selected as test samples. Two grams of each sample was mixed with 10 mL of deionized water in a centrifuge tube. In order to extract maltol and ethyl maltol sufficiently from a sample, the mixture was homogenized (6000 rpm) for 3 min with a high-speed homogenizer (XHF-DY, Scientitz, 84 Ningbo, China), and then treated with ultrasound for 10 min (SB-800DT, Ningbo, China). After ultrasonic treatment, the sample aqueous solution was centrifuged (4000 rpm) for 5 min. The supernatant was collected, and then further treated using the DLLME technique.

Food matrix components (such as protein and fatty) interact with organic solvent in DLLME to cause an emulsifying phenomenon, which reduces the volume of the separated organic phase [31]. In DLLME, high preconcentration factor and sufficient volume of precipitated phase must be ensured for further analysis after centrifugation. Generally, the volume of dispersant and extractant are in the ranges of 50–3000 µL and 15–2000 µL, respectively [31]. In this study, extractant (500 µL) and dispersant (1.5 mL) were mixed, and then injected into the sample supernatant (2 mL). After centrifugation (6000 rpm) for 3 min, 200 μL of the lower organic phase mixed with 15 µL of cyclohexanone standard (50 mg/L) was transferred into 20 mL headspace vials and further evaporated under vacuum conditions to eliminate the organic solvent. The dried sample was incubated at a constant temperature, and then further extracted by head-space SPME (DVB/CAR/PDMS, 50/30 m). In this study, the optimal conditions for the DLLME treatment were selected by comparing the effects of different extractants (dichloromethane, chloroform, and carbon disulfide), dispersants (methanol, acetonitrile, and acetone), extractant-to-dispersant volume ratios (1:4, 2:5, and 1:2), and ratios of water sample volume to total volume of dispersant and extractant (2:3, 4:5, and 1:1) on the extraction effect of flavor enhancers. The optimal conditions for the HS-SPME treatment were selected by comparing the results of different incubation temperatures (40 °C, 50 °C, and 60 °C), incubation times (10 min, 15 min, 20 min, and 25 min), and extraction times (10 min, 20 min, 30 min, and 40 min) on flavor enhancer extraction.

### 2.3. GC-MS Determination of Flavor Enhancers

The flavor substances in the samples were separated using a 7890B series gas chromatograph, and the target substances were quantified using a 5977B series mass spectrometer. Chromatographic conditions of the 7890B were: the chromatographic column was an Agilent 19091S-431UI-5MS capillary column (15 m × 250 μm × 0.25 μm), the sample injection volume was 1 μL, and the injection port temperature was 250 °C. The temperature rise program was 35 °C, containing for 0 min, rising to 220 °C at 5 °C /min, and then, rising to 280 °C at 10 °C /min and containing for 2 min. The carrier gas was high purity helium (99.9%), the flow rate was 1 mL/min, the column pressure was 12.04 psi, and the injection port was in the undivided mode. The GC column was directly connected to an Agilent 5977 B series mass selective detector of ion source for the mass spectrometry analysis. The EI source was used as the ion source, the analyte was ionized in the ion source at 70 eV and 230 °C, and the scanning mass range was 40–400 amu [32].

The matrix effect (ME) in samples was calculated using the following equation:

(1) $$\mathrm{ME}\%\;=\frac{\;\mathrm{Peak}\;\mathrm{area}\;\mathrm{of}\;\mathrm{standards}\;\mathrm{in}\;\mathrm{matrix}-\mathrm{Peak}\;\mathrm{area}\;\mathrm{of}\;\mathrm{standards}\;\mathrm{in}\;\mathrm{solvent}}{\mathrm{Peak}\;\mathrm{area}\;\mathrm{of}\;\mathrm{standards}\;\mathrm{in}\;\mathrm{solvent}}\text{×}100\%$$

### 2.4. Statistical Analysis

The mean and standard deviation of each experiment were calculated. Analysis of variance (ANOVA) was used to determine significant differences (*p* < 0.05) between each experiment using the SPSS software package (IBM SPSS Statistics 20).

## 3. Results and Discussion

### 3.1. Optimization of the DLLME/HS-SPME Conditions

**Figure 1:**
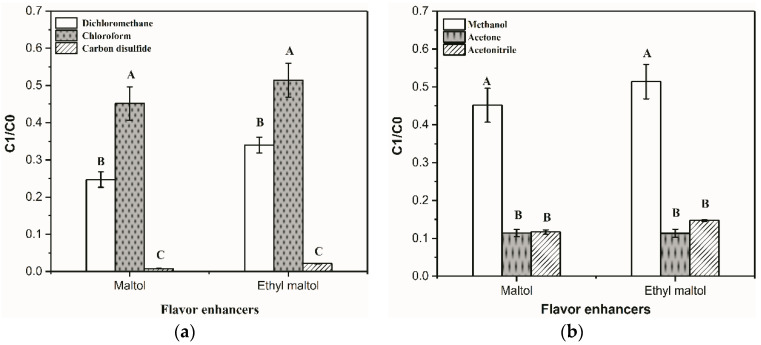

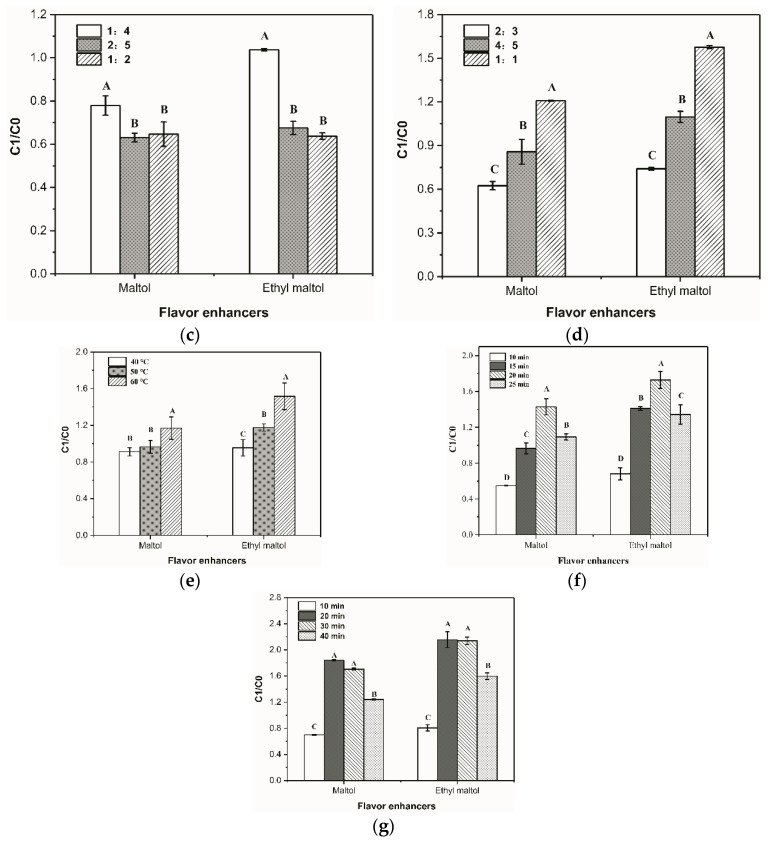
Optimization of conditions for pretreatment dispersive liquid-liquid microextraction/headspace solid-phase microextraction (DLLME/HS-SPME): (**a**) Different types of extractants (dichloromethane, chloroform, and carbon disulfide); (**b**) different types of dispersants (methanol, acetonitrile, and acetone); (**c**) different extractant volume to dispersant volume ratios; (**d**) different aqueous volume to total volume of dispersant and extractant; (**e**) different incubation temperatures (40 °C, 50 °C, and 60 °C); (**f**) different incubation times (10 min, 15 min, 20 min, and 25 min); (**g**) different extraction times (10 min, 20 min, 30 min, and 40 min), on the extraction efficiency of flavor enhancers. Note: C1/C0, the peak area of flavor enhancers (maltol and ethyl maltol)/the peak area of cyclohexanone standard. The superscript letters (A–D) in each histogram indicate significant differences (*p* < 0.05) for the samples.

To reduce the pretreatment time and to reduce the matrix effect on target compounds, DLLME was used to eliminate matrix interference quickly and to improve the method’s sensitivity before the HS-SPME/GC-MS analysis. In this study, the conditions of DLLME were optimized using a single factor design experiment. The maltol and ethyl maltol in the water phase were extracted by organic solvent. As shown in Figure 1a,b, the extraction efficiency was increased when chloroform was performed as an extractant and methanol were performed as a dispersant. Therefore, chloroform and methanol were the best extractant and dispersants for DLLME in this experiment. Chloroform is the most widely used extractant (approximately 42% of published studies use this solvent) [31].

Similarly, the ratio of each phase in the solution would also affect the extraction efficiency of target analytes. As shown in Figure 1c, the highest extraction efficiency of maltol and ethyl maltol was obtained at 1:4 (the volume ratio of extractant to dispersant). When the volume ratio of extractant to dispersant in the organic phase increased, the extraction efficiency of maltol and ethyl maltol correspondingly decreased due to the dilution effect. In addition, a decrease in organic phase volume ratio could effectively increase the extraction efficiency of maltol and ethyl maltol (Figure 1d). However, the lower volume ratio of organic phase made it difficult to obtain the separated organic phase with enough volume to pipette out. In this study, the volume ratio of the sample (2 mL) to the organic phase was selected as 1:1.

The extraction time, incubating time, and extraction temperature in the HS-SPME method were separately optimized. As shown in Figure 1e, the HS-SPME method was most suitable for removing maltol and ethyl maltol from the DLLME extract when the extraction temperature was 60 °C. Increasing the extraction temperature significantly influenced the extraction of flavor substances. An increase in temperature could increase the content of volatile compounds in the headspace, and therefore, increase extraction efficiency, but excessive high temperature (>60 °C) could induce the desorption of volatile flavor compounds on HS-SPME fiber [32]. Therefore, the best extraction temperature was set at 60 °C. In addition, the incubation time and extraction time had significant effects on the extraction efficiency of HS-SPME. When the incubation time (Figure 1f) and extraction time (Figure 1g) were 20 min separately, the highest extraction efficiency of maltol and ethyl maltol were obtained.

### 3.2. Verification of Pretreatment Effect of DLLME

**Figure 2:**
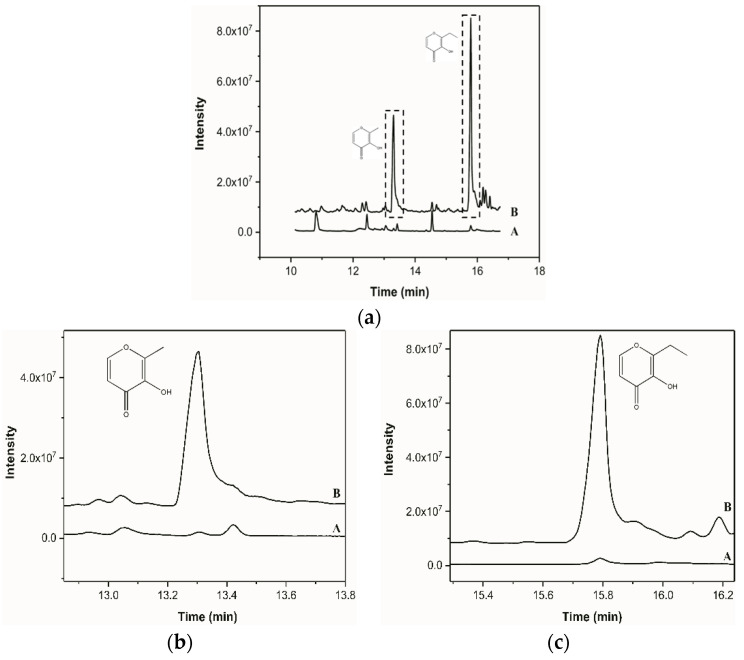
Chromatograms of maltol and ethyl maltol pretreated by dispersive liquid-liquid microextraction combined with headspace solid phase microextraction (A, without DLLME treatment and B, with DLLME treatment): (**a**) Chromatograms of maltol and ethyl maltol standard; (**b**) enlargement of the chromatogram of maltol standard; (**c**) enlargement of the chromatogram of ethyl maltol standard.

**Figure 3:**
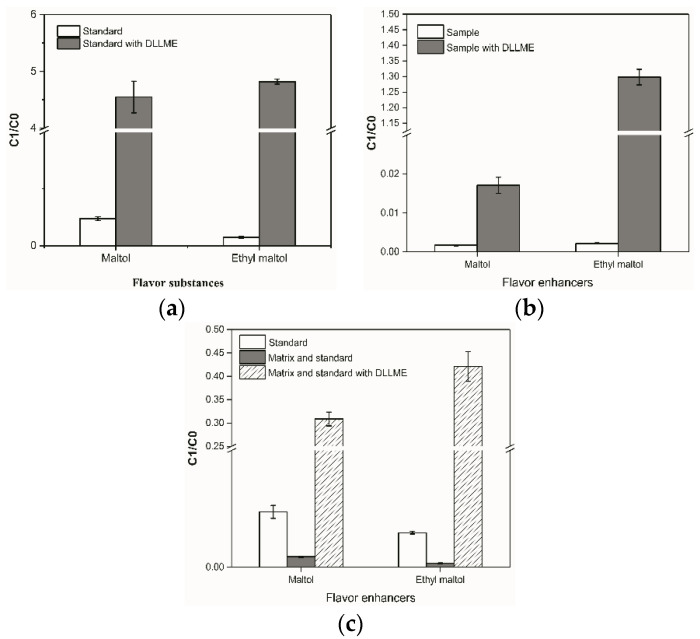
Effects of different pretreatments (without DLLME treatment and after DLLME treatment) on extraction efficiency of maltol and ethyl maltol: (**a**) Effect of DLLME treatment on the extraction efficiency of maltol and ethyl maltol standards; (**b**) effect of DLLME treatment on the extraction efficiency of maltol and ethyl maltol in the matrix (dried squid samples); (**c**) effect of DLLME treatment on the extraction efficiency of maltol and ethyl maltol in the matrix with standards. C1/C0, the peak area of flavor enhancers (maltol and ethyl maltol)/the peak area of cyclohexanone standard.

In this study, the DLLME/HS-SPME/GC-MS method was evaluated with 1 μg/mL maltol and ethyl maltol standards. As shown in Figure 2a, HS-SPME/GC-MS could effectively detect volatile maltol and ethyl maltol. The chromatographic peaks obtained were well separated (retention time was 13.3 min and 15.8 min, respectively) with symmetrical peak shapes. As compared with the HS-SPME method, the signal intensity of maltol (five times increase) and ethyl maltol (10 times increase) was improved significantly when DLLME was coupled with HS-SPME (Figure 3a).

Complex matrix samples such as seafood contain a high content of oil and protein [33], which significantly interfere with HS-SPME enrichment of maltol and ethyl maltol. Obviously, the signal intensity of maltol and ethyl maltol with sample matrix was individually reduced by five and nine times as compared with the samples without matrix (Figure 3c). However, the signal intensity of maltol and ethyl maltol with matrix was significantly improved after DLLME pretreatment prior to HS-SPME (Figure 3b).

The calculated matrix effect in seafood was −81.12% for maltol and −88.72% for ethyl maltol (Table 1). The enrichment factor of the DLLME method coupled with HS-SPME was 19 for maltol and 66 for ethyl maltol (Table 1). The results showed that DLLME improved the efficiency of the HS-SPME/GC-MS analysis of volatile flavor compounds.

### 3.3. Evaluation of the Analytical Method

The applicability of DLLME combined with HS-SPME/GC-MS in the analysis of flavor enhancers (maltol and ethyl maltol) in seafood was evaluated based on linear range, sensitivity, stability, and accuracy. The standard curves of maltol and ethyl maltol were obtained by adding maltol standard (0.25–25 μg/g) and ethyl maltol standard (0.05–40 μg/g) into dried squid substrate. As shown in Table 1, the method had an excellent linear relationship, and the correlation coefficients (R^2^) of the calibration curves for maltol and ethyl maltol were greater than 0.995. In this study, the limit of detection (LOD) values of maltol and ethyl maltol were 5.0 μg/kg and 2.5 μg/kg, respectively. In addition, the limit of quantification (LOQ) values were 15 μg/kg and 5.0 μg/kg, respectively. As compared with the previously reported methods of solid-phase extraction and dispersed liquid-liquid microextraction (DSPE-DLLME), ionic liquid (IL), and solid-phase extraction (SPE), the LOD and LOQ of the developed method were relatively low (Table 2). Obviously, DLLME coupled with HS-SPME was sensitive for determining maltol and ethyl maltol in complex matrix samples.

The relative standard deviation (RSD) was detected to evaluate the stability and repeatability of the DLLME/HS-SPME pretreatment method. Intraday precision and interday precision were determined by adding a 5.0 μg/g mixed standard solution of maltol and ethyl maltol to dried squid samples. As shown in Table 1, the intraday accuracy ranged from 2.8 to 5.7% and the interday accuracy ranged from 3.5 to 4.3% for the DLLME/HS-SPME method. The results indicated that the developed method was stable and reliable.

The recovery rate of this method was evaluated by adding three different concentrations of maltol and ethyl maltol standard solutions to dried squid samples. The recovery results showed that the pretreatment of instant dried squid by the DLLME/HS-SPME method did not affect the accuracy of GC-MS detection of flavor substances. As shown in Table 3, the recovery rate of maltol in matrix samples ranged from 89.0 to 118.6%, and that of ethyl maltol in matrix samples ranged from 96.0 to 112.1%. The results suggested the developed method was accurated for determining maltol and ethyl maltol in complex matrix samples.

### 3.4. Determination of Maltol and Ethyl Maltol in Authentic Seafood

Four kinds of seafood (squid larvae, dried squid, seasoned kelp, and crispy yellow croaker) were pretreated by DLLME, and then further detected and analyzed by HS-SPME/GC-MS. As shown in Table 4, the contents of maltol and ethyl maltol in four kinds of seafood ranged from 0.7 to 2.2 μg/g and from 0.9 to 34.7 μg/g, respectively. The results showed that the method was suitable for the detection and analysis of flavor enhancers in marine products. According to the EU legislation, the added concentration of maltol to food can range from 50 to 200 mg/kg [37]. The recommended maximum acceptable daily dose for the human body is 2 mg/kg [1]. The addition amounts of maltol and ethyl maltol in squid larvae, dried squid, seasoned kelp, and crispy yellow croaker were within the maximum allowable addition range (200 mg/kg).

## 4. Conclusions

In this study, flavor enhancers (maltol and ethyl maltol) in solid seafood were detected by using DLLME combined with an HS-SPME/GC-MS analysis. The developed method could significantly eliminate the matrix effect and could significantly improve the method’s sensitivity. As expected, DLLME effectively broadened the HS-SPME applications for volatile compounds determination in complex samples.

## Figures and Tables

**Table 1:** Calibration range, limit of detection (LOD), and limit of quantitation (LOQ) for flavor enhancers.

Compounds	Maltol	Ethyl Maltol
Matrix effect (%)	−81.12%	−88.72%
Enrichment factor	19	66
Calibration range (μg/g)	0.25–25.00	0.05–40.00
Regression equation ^a^	*y* = 0.2806*x* − 0.0142	*y* = 0.6744*x* + 0.2082
R^2^	0.9975	0.9967
LOD (μg/kg) ^b^	5.0	2.5
LOQ (μg/kg) ^c^	15.0	5.0
Intraday precision RSD (*n* = 3, %)	5.7	2.8
Interday precision RSD (*n* = 3, %)	4.3	3.5

^a^ *y* is the peak area of flavor substances and *x* is the concentration of flavor substances; ^b^ S/N = 3; ^c^ S/N = 10.

**Table 2:** Comparison of the present method with other methods ^a^.

Detection Methods	Matrix	Analytes	LOD	LOQ	Reference
DSPE/DLLME-HPLC-PDA	Ready-to-eat seafood	Flavor enhancers (maltol, ethyl maltol, vanillin, methyl vanillin, ethyl vanillin)	60–150 μg/kg	200–500 μg/kg	[34]
IL-IC	Biscuit, chocolate, and milk powder	Spices (vanillin, ethyl vanillin and ethyl maltol)	20–45 μg/kg	70–150 μg/kg	[35]
SPE-GC–MS	Infant formula	Flavoring agents (vanillin, methyl vanillin, ethyl vanillin and coumarin)	-	10 µg/kg	[36]
DLLME/HS-SPME/GC-MS	Seafood	Flavor enhancers (maltol, ethyl maltol)	2.5–5.0 µg/kg	5–15 µg/kg	This work

^a^—not mentioned.

**Table 3:** Recovery of maltol and ethyl maltol in samples ^a^.

Flavor Enhancers	Spiking Level (μg/g)	Recovery (RSD%)
Maltol	1.00	89.0 (4.2)
	12.50	98.1(4.8)
	20.00	118.6 (3.3)
Ethyl maltol	2.50	106.1 (6.8)
	12.50	96.0 (1.1)
	25.00	112.1 (4.4)

^a^ Results shown represent % recovery with % RSD in parentheses, *n* = 3.

**Table 4:** Determination of maltol and ethyl maltol in seafood ^a^.

Sample	Maltol (μg/g)	Ethyl Maltol (μg/g)
Squid larvae	0.7 (5.5)	1.1 (9.0)
Dried squid	1.4 (8.0)	34.7 (7.5)
Seasoned kelp	2.2 (4.6)	5.3 (3.8)
Crispy yellow croaker	0.7 (8.8)	0.9 (7.1)

^a^ % RSD values were given in parentheses, *n* = 3.

## Data Availability

The data that support the findings of this study are available from the corresponding author upon reasonable request.
